# Integrating gold nanoclusters, folic acid and reduced graphene oxide for nanosensing of glutathione based on “turn-off” fluorescence

**DOI:** 10.1038/s41598-021-81677-8

**Published:** 2021-01-27

**Authors:** Xin Yi Wong, Daniel Quesada-González, Sivakumar Manickam, Siu Yee New, Kasturi Muthoosamy, Arben Merkoçi

**Affiliations:** 1 Department of Chemical and Environmental Engineering, Faculty of Science and Engineering, University of Nottingham Malaysia, 43500 Semenyih, Selangor Malaysia; 2 Paperdrop Diagnostics, Av. de Can Domènech s/n, Eureka Building, Campus UAB, 08193 Bellaterra, Barcelona, Spain; 3 Nanotechnology Research Group, Centre of Nanotechnology and Advanced Materials, University of Nottingham Malaysia, 43500 Semenyih, Selangor Malaysia; 4 Petroleum and Chemical Engineering, Faculty of Engineering, Universiti Teknologi Brunei, Bandar Seri Begawan, BE1410 Brunei Darussalam; 5 School of Pharmacy, Faculty of Science and Engineering, University of Nottingham Malaysia, 43500 Semenyih, Selangor Malaysia; 6 Nanobioelectronics and Biosensors Group, Catalan Institute of Nanoscience and Nanotechnology (ICN2), CSIC, Barcelona, Spain; 7 The Barcelona Institute of Science and Technology (BIST), Campus UAB, 08036 Bellaterra, Barcelona, Spain; 8 ICREA, Institució Catalana de Recerca i Estudis Avançats, Pg. Lluis Companys 23, 08010 Barcelona, Spain

**Keywords:** Nanoscience and technology, Biomaterials, Nanobiotechnology

## Abstract

Glutathione (GSH) is a useful biomarker in the development, diagnosis and treatment of cancer. However, most of the reported GSH biosensors are expensive, time-consuming and often require complex sample treatment, which limit its biological applications. Herein, a nanobiosensor for the detection of GSH using folic acid-functionalized reduced graphene oxide-modified BSA gold nanoclusters (FA-rGO-BSA/AuNCs) based on the fluorescence quenching interactions is presented. Firstly, a facile and optimized protocol for the fabrication of BSA/AuNCs is developed. Functionalization of rGO with folic acid is performed using EDC/NHS cross-linking reagents, and their interaction after loading with BSA/AuNCs is demonstrated. The formation of FA-rGO, BSA/AuNCs and FA-rGO-BSA/AuNCs are confirmed by the state-of-art characterization techniques. Finally, a fluorescence turn-off sensing strategy is developed using the as-synthesized FA-rGO-BSA/AuNCs for the detection of GSH. The nanobiosensor revealed an excellent sensing performance for the detection of GSH with high sensitivity and desirable selectivity over other potential interfering species. The fluorescence quenching is linearly proportional to the concentration of GSH between 0 and 1.75 µM, with a limit of detection of 0.1 µM under the physiological pH conditions (pH 7.4). Such a sensitive nanobiosensor paves the way to fabricate a “turn-on” or “turn-off” fluorescent sensor for important biomarkers in cancer cells, presenting potential nanotheranostic applications in biological detection and clinical diagnosis.

## Introduction

Glutathione (GSH) is a low molecular weight (307.32 g mol^−1^) tri-peptide composed of glutamate, cysteine and glycine^1–3^. As an endogenous antioxidant, GSH may scavenge reactive oxygen species, protect cells from oxidative stress and subsequently inhibit cancer progression^1,2,4–7^. GSH levels are found to be elevated in illness/cancers such as Alzheimer’s disease^8^ as well as ovarian^9^, liver^3^, lung, colorectal, breast, head and neck cancer patients^10^. Reducing intracellular GSH has been proposed as one of the strategies of cancer treatment^11,12^. Hence, sensing of GSH in the biological samples for diagnosis of diseases is of great interest in biological applications.

Although commercial kits for the detection of GSH are available, there is still a need for a detection platform with the advantages of lower cost, fast response time, more sensitive and essentially stable^13^. The use of nanomaterials in biosensing is becoming popular in recent years due to the improvements in sensitivity and its robustness^14–17^ since contrary to enzymes and other biological compounds, nanomaterials are stable in time and do not require, in most of the cases, special storage conditions (i.e. low temperature, buffered medium, etc.). In the recent decade, polydopamine nanoparticles (NPs)^18^, iron pyrite NPs^19^, carbon dots^20^ and mixed-valence-state cobalt nanomaterials^21^ have been designed for GSH sensing. Particularly, manganese dioxide (MnO_2_) nanosheets are usually selected as GSH biosensors, owing to their excellent colloidal stability, absorption capability, redox chemistry and biocompatibility^22–24^. However, toxicity studies of MnO_2_ nanosheets in the biological environment are still in the preliminary stage^25,26^. Although previous reports on GSH sensing using fluorescence methods were claimed as cost-effective and highly sensitive, however, those methods involve complicated experimental procedures and are time-consuming^27–29^.

The size of gold nanoparticles (AuNPs) is in the range of 3–100 nm, which is close to the wavelength of light^30^. The collective oscillation of free conduction band electrons on the surface of AuNPs can interact with electromagnetic waves and generate localised surface plasmon resonance (SPR) effect^31^. The SPR contributes to the optical and electronic properties of AuNPs. In contrast, gold nanoclusters (AuNCs) are groups of several to a few hundreds of gold atoms with a size smaller than 3 nm^32^. AuNCs have dimensions between those of atoms and metallic NPs^33^. The size of AuNCs is comparable to the Fermi wavelength of conduction electrons^34–36^. Although the size of AuNCs is too small to support the SPR effect, AuNCs exhibit unique size-dependent fluorescence and molecule-like optical properties^30^, owing to the ultrasmall size and corresponding electronic structure. Aside from Au cores, AuNCs are protected by organic ligand shells and stabilised by thiol groups of the ligand. The emission relies on the conformation of a ligand shell and core, as well as ligand-to-metal charge transfer states^31^. Therefore, ligand engineering, such as modifying the surface ligands of AuNCs with functional groups, is crucial in regulating the optical properties of AuNCs^37^.

AuNCs are emerging nanomaterials with diverse applications in the biomedical field. AuNCs display discrete energy levels, large Stokes shift, good water solubility, distinctive fluorescence properties, high photostability and biocompatibility^32,38,39^. These features make AuNCs a perfect candidate for imaging, biosensing and theranostics in the cellular and molecular level^40,41^. Functional graphene oxide (GO) has been proven to be a viable carrier of drugs, imaging and therapeutic agent owing to its high loading capacity, strong adsorption capacity for serum proteins, small size, intrinsic optical properties and large surface area^41–43^. Functional GO has two aromatic planes and is capable of adsorbing aromatic compounds via simple physisorption (mainly π–π stacking and hydrophobic interactions)^42^. To our knowledge, many studies have been carried out in loading of cancer drugs, such as doxorubicin and camptothecin, onto GO functionalised with an antibody for theranostic of cancer cells^42,43^. Particularly, reduced graphene oxide (rGO) is a biocompatible drug delivery carrier, a photothermal and bioimaging agent with potential in theranostics of cancer/disease/illness^41,44–50^. And finally, folic acid (FA) is a water-soluble vitamin B9, widely used in drug targeting owing to its low-cost, compatibility in both organic and aqueous solvents and lack of immunogenicity^51^. FA is used for active targeting of folate receptors on cancer cells.

Sensing of FA based on the fluorescence quenching of BSA/AuNCs has been introduced by Hemmateenejad et al.^52^. It is speculated that FA interacts via hydrophilic and hydrophobic interactions with tryptophan residues (Trp-132 on the surface and Trp-212 residue inside) of BSA. The interaction of FA and BSA alters BSA protein secondary structure, causing a partial protein unfolding and therefore results in fluorescence quenching^53^. Although the sensor previously reported can be applied in the determination of FA in pharmaceutical preparations, its application as a delivery carrier remains unexplored till date.

Conjugation of FA on graphene-based nanosystem can target and induce higher cytotoxicity on folate receptor- positive cells such as breast, ovarian, lung and colon cancers^54–56^. FA plays a dual role (as a reductant and stabilizer) in FA-modified rGO (FA-rGO). The covalent binding of FA to rGO can produce stable and biocompatible materials^57^, enhance the energy accepting efficiency in long-range resonance energy transfer process than graphene^58^, thereby making it suitable to be employed as a biosensor or drug delivery carrier. Therefore, conjugation of FA to rGO serves as a potential nanocarrier for controlled loading and targeted delivery of therapeutic agents^42,59^.

Despite its remarkable promise, no reports have been devoted to the construction of a fluorescent sensing platform with FA-rGO and bovine serum albumin-templated AuNCs (BSA/AuNCs). The effect of GSH on the FA-rGO-BSA/AuNCs has also remained largely unexplored. There are a few reports that proposed the use of BSA/AuNCs-Cu system for GSH sensing^47,48^, in which Cu^2+^ is used as a fluorescence quencher for BSA/AuNCs, and the fluorescence can be recovered with the addition of GSH. However, this is the first work carried out in exploring the potential of FA-rGO not only as a carrier of BSA/AuNCs but also having the potential as a GSH nanobiosensor with significantly improved sensitivity and selectivity. The elucidation of such mechanism will enable us to design effective fluorescent sensors for targets of interest rationally. In this investigation, we aim at understanding the quenching mechanism of FA-rGO towards BSA/AuNCs fluorescence, which in turn allows us to design effective turn-off fluorescent nanosensors for GSH detection. This is the first time that BSA/AuNCs, FA and rGO are combined for fluorescent sensing of GSH, with a simple experimental process that requires short incubation time of only 2 min.

## Experimental

### Reagents and materials

Bovine serum albumin (96%, BSA, Sigma) was purchased in lyophilized-powder form and used without further purification. Gold (III) chloride solution (HAuCl_4_), folic acid (> 97%), reduced l-glutathione (≥ 98%), *N*-hydroxysuccinimide (NHS), *N*-(3-dimethylaminopropyl)-*N*′-ethylcarbodiimide hydrochloride (EDC), and all other reagents were purchased from Sigma Aldrich and used as received. Ultrapure deionized water was obtained from a Milli-Q Plus system (EMD Millipore, Billerica, MA, USA). Sodium hydroxide (1 M, NaOH) was purchased from Nacalai Tesque. l-ascorbic acid was purchased from R&M Chemicals. Reduced graphene oxide (rGO) was synthesized using our previously reported protocol^60^.

### Analytical measurements

Fluorescence spectra were recorded using a fluorescence spectrophotometer (Hitachi F-7000). UV–Vis absorbance was measured using UV–Vis spectrophotometer (Lambda 35, Perkin Elmer) to ensure the absence of large NPs, which commonly show absorption at about 520 nm. UV light with the excitation of 365 nm was used. To study the protein conformation, far-UV circular dichroism (CD, J-1000 series, JASCO) was employed. The oxidation state of core Au atoms was examined by X-ray photoelectron spectroscopy (XPS, ULVAC-PHI, Inc.). The morphological characterization of BSA/AuNCs was carried out using a high-resolution transmission electron microscope (HRTEM, FEI Tecnai G^2^ F20 X-Twin). The Fourier transform infrared spectroscopy (FTIR) spectra of FA, rGO and FA-rGO were recorded on a FTIR spectrometer (PerkinElmer Frontier).

### Synthesis of BSA/AuNCs

BSA/AuNCs were synthesized following a modified protocol of Xie et al.^61^. Briefly, 0.7 mL of 12 mM HAuCl_4_ solution was added to the same amount of aqueous solution containing 20 mg mL^−1^ BSA in a thermomixer and mixed at 1200 rpm for 5 min at 40 °C. Then, 0.1 mL of 1 M NaOH solution was introduced, and the mixture was mixed in the thermomixer at 900 rpm for 6 h at 60 °C. The color of the solution changed from light yellow to deep brown, which indicates the successful synthesis of BSA/AuNCs. The resulting solution was purified using EMD Millipore Amicon Ultra-0.5 centrifugal filter units with a membrane molecular weight cut-off (MWCO) of 10 kDa were used to remove residual ions (i.e. Na^+^, Au^3+^ and OH^−^). The products were then stored at 4 °C until further use.

### Covalent conjugation of FA-rGO

FA-rGO was prepared using a modified protocol reported by Zhang et al.^42^. Briefly, 1 mg mL^−1^ rGO was subjected to probe sonication of 20 kHz, at 500 W for 10 min. NaOH (6.25 mmol) and chloroacetic acid (0.250 g, 11.655 mmol) was then added. The mixture was bath sonicated (40 kHz, 70 W) for 2 h. After neutralization with HCl, the mixture was purified by repeated rinsing and centrifugation until rGO is well dispersed in deionized water. The mixture was dialyzed against deionized water for 24 h. To introduce sulfonate groups to the rGO, sulfanilic acid (51.96 mg, 0.06 M) and sodium nitrite (70.720 mg, 0.205 M) were dissolved in 20 mL of 0.25 v/v % 1 M NaOH. The solution was added dropwise to 0.1 M HCl in an ice bath. The sulfonated groups were mixed with rGO in an ice bath under stirring for 2 h, followed by dialysis against deionized water for over 24 h. The mixture was stored at 4 °C until further use. EDC and NHS were added onto rGO, with the molar ratio of rGO:EDC:NHS as 40:50:73. The mixture was subjected to probe sonication for 2 h. FA (5 mg mL^−1^, dissolved in 0.5 M NaHCO_3_, at pH 8) was added and stirred overnight. The products were dialyzed against 0.5 M NaHCO_3_ for 24 h, followed by dialysis against deionized water for over 24 h. The products were characterized by FTIR, UV–Vis spectroscopy and fluorescence spectrophotometer.

### Fluorescence quenching of BSA/AuNCs by FA-rGO

To investigate the potential of FA-rGO to induce fluorescence quenching of BSA/AuNCs, different concentrations of FA-rGO were added to 3 mg mL^−1^ of BSA/AuNCs. The solution was mixed in a thermomixer at 1200 rpm for 5 min at room temperature. Fluorescence intensity of the solution was recorded with an excitation wavelength (λ_ex_) of 365 nm.

### Sensing of GSH

GSH detection was conducted as follows. The same volume of FA-rGO-BSA/AuNCs was added with various concentrations of GSH. The solution was mixed in a thermomixer at 1200 rpm for 2 min at room temperature. The fluorescence intensity was measured to quantify the concentration of GSH at λ_ex_ = 365 nm.

### Calculation of the signal-to-noise ratio and limit of detection

Signal-to-noise ratio (SNR) was calculated using the following standard Eq. (1)

1 $$SNR = R_L/S_1$$

Limit of detection (LOD) was calculated according to the following standard Eqs. (2) and (3)

2 $$y = a + SX$$

here, X is LOD

3 $$y = (K \times S_1) + Blank$$

wherein,

$$a + (S + LOD) = (K \times S_1) + Blank$$

Therefore,

$$LOD = {{\left[ {(K \times S_1) + Blank - a} \right]} \mathord{\left/ {\vphantom {{\left[ {(K \times S_{1}) + Blank - a} \right]} S}} \right. \kern-\nulldelimiterspace} S}$$

where R_L_ is the signal response of least known concentration, K is the coefficient 3.3^62^, S is the slope obtained from a calibration curve using Fig. 5, while S_1_ is the statistical result of the standard deviation of the blank solution and *a* is the blank value.

## Results and discussion

### Synthesis and characterization of BSA/AuNCs

Different fabrication methods of protein-templated AuNCs have been proposed since the year 2009; however, the long reaction time (up to 12 h), low quantum yields (QYs) (about 6%) and complicated protocols are some of the existing limitations. In this work, a simple protocol for the fabrication of BSA/AuNCs with several advantages has been reported. The advantages of this protocol are shorter synthesis time (only 6 h), higher QYs (10.62%), need for lower protein amount (only 20 mg mL^−1^); and employ mild reaction conditions. The protocol is also applicable for the fabrication of AuNCs with different protein templates such as lysozyme or ribonuclease A (RNase A, as tested, data not shown), and not limited to BSA alone. Overall, such a synthesis protocol is more economical and eco-friendly.

**Figure 1:**
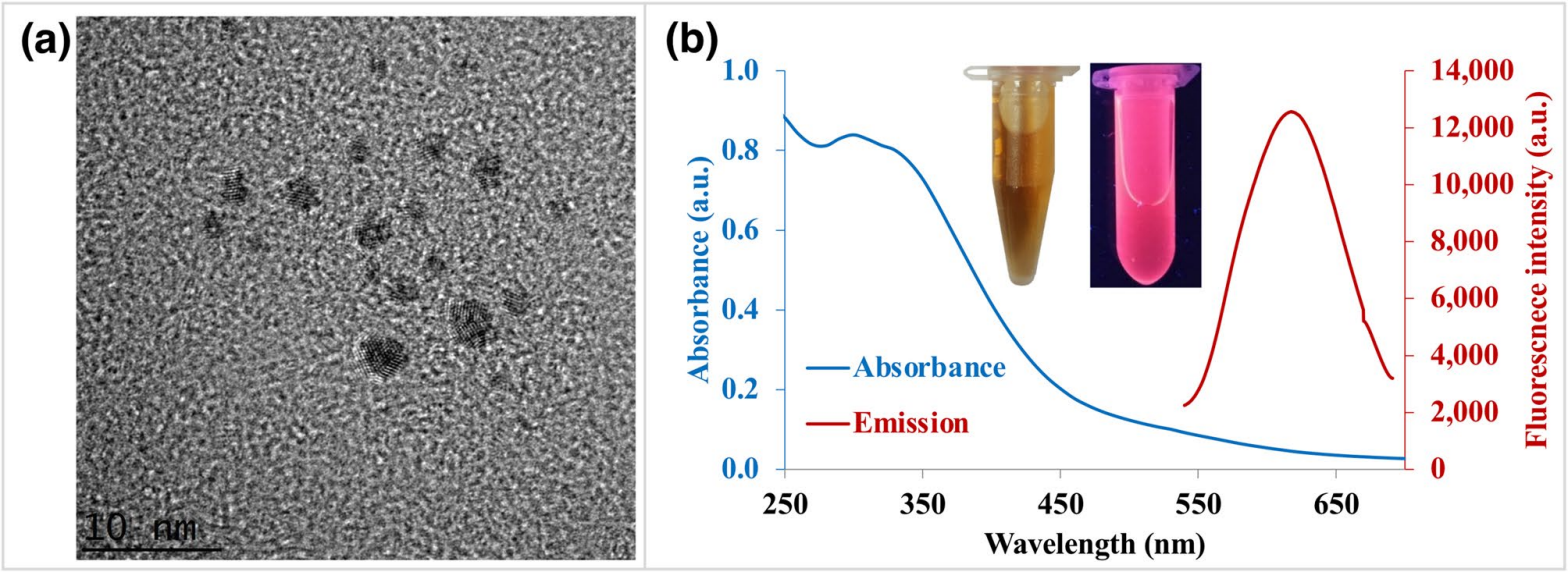
Characterization of BSA/AuNCs. (**a**) HRTEM image of the diluted fluorescent BSA/AuNCs. (**b**) UV–Vis absorption (blue line) and fluorescence emission spectra (red line) of the as-prepared BSA/AuNCs with λ_ex_ at 365 nm. Inset: optical photographs of the BSA/AuNCs under visible (left) and UV light (right).

HRTEM image (Fig. 1a and Supplementary Fig. S1) demonstrated that the sizes of the BSA/AuNCs fall within a narrow range of less than 2 nm. BSA/AuNCs are generally spherical dots demonstrating uniform size with high mono-dispersity. As shown in the optical absorption of the as-prepared BSA/AuNCs (Fig. 1b), no apparent SPR absorption peak could be observed in the range between 400 and 600 nm^34^. The absorption of BSA/AuNCs monotonously increases towards the shorter wavelength over the range of 220–850 nm. These confirm the encapsulation of AuNCs in BSA protein, and most importantly, no large NPs (> 2 nm in diameter) were formed^63^ when excited at 514 nm. The obtained QYs is higher than the previously reported value of around 6%^61^. The earlier study has shown that BSA starts to unfold at 65 °C^64^; therefore, a temperature of 60 °C was chosen for the synthesis, instead of the physiological temperature (37 °C). This is because, upon heating, the compact native form of BSA becomes more flexible and reactive, exposing the Tyr and Trp residues from the hydrophobic core of BSA molecule to a more polar solvent environment^64,65^. A higher interaction between BSA and Au ions fastens the formation of BSA/AuNCs.

**Figure 2:**
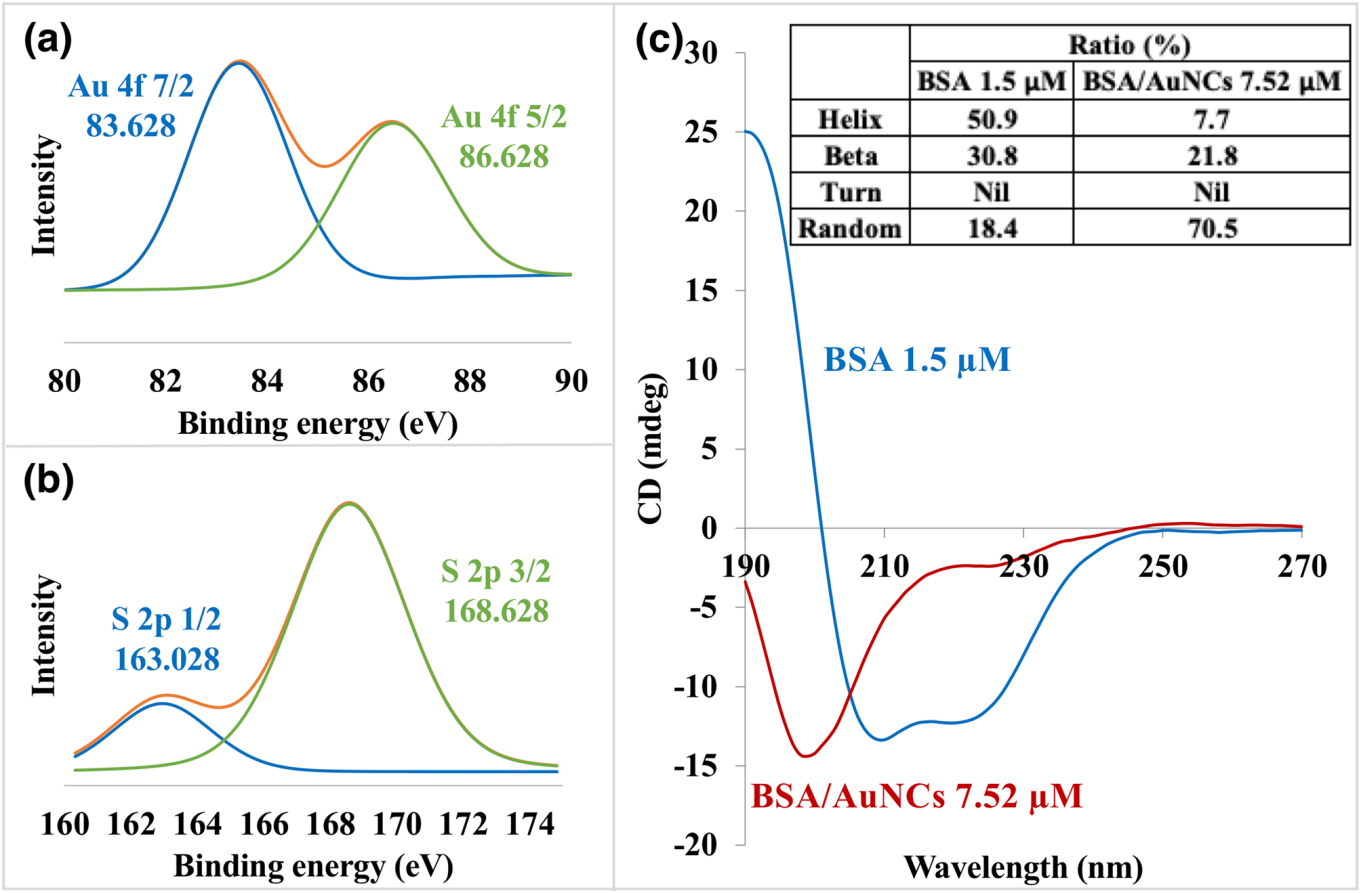
Characterization of BSA/AuNCs. Representative X-ray photoelectron spectroscopy (XPS) spectra of (**a**) Au 4f, (**b**) S 2p and (**c**) CD spectra of BSA/AuNCs.

XPS was employed to investigate the protein-AuNCs interactions and to prove the reducibility of the protein against Au(III) ions in alkaline pH (Supplementary Fig. S2). As shown in Fig. 2a, the Au 4f of XPS and the binding energies at 83.628 eV (Au 4f_7/2_) and 86.628 eV (Au 4f_5/2_) confirm the formation of stable BSA/AuNCs, with most of the Au atoms close to the oxidation state of Au(0). The two S 2p bands with the binding energies of about 163 (S 2p_1/2_) and 168 eV (S 2p_3/2_) were observed (Fig. 2b), corresponding to the gold-bound (Au–S) and oxidized sulfur species, respectively. Their relevant abundances were estimated as 48.5 and 51.5%, respectively, from the XPS curve fit of BSA/AuNCs.

CD spectroscopy was employed to further investigate the conformational evolution of native and AuNCs-bound proteins. From the CD spectra of BSA/AuNCs (Fig. 2c), the characteristics of the two negative bands of the typical α-helix at 208 and 220 nm were observed. This corresponds to π to π* and n to π* transitions, due to the peptide bond of an α-helix. Attributed to the nucleation of AuNCs, the intensity of α-helix peaks shows a gradual declination with the addition of Au. It shows 85% reduction in the α-helix with a 30% increase in the β-sheet after the synthesis of BSA/AuNCs. Therefore, it can be deduced that the interaction between these molecules are complex and cause multidirectional alterations in the structure of the protein. The minimum observed at 220 nm shifts towards lower wavelength, indicating a steady increase in the content of disordered structures in the BSA of AuNCs^66^.

### Characterization of FA-rGO

**Figure 3:**
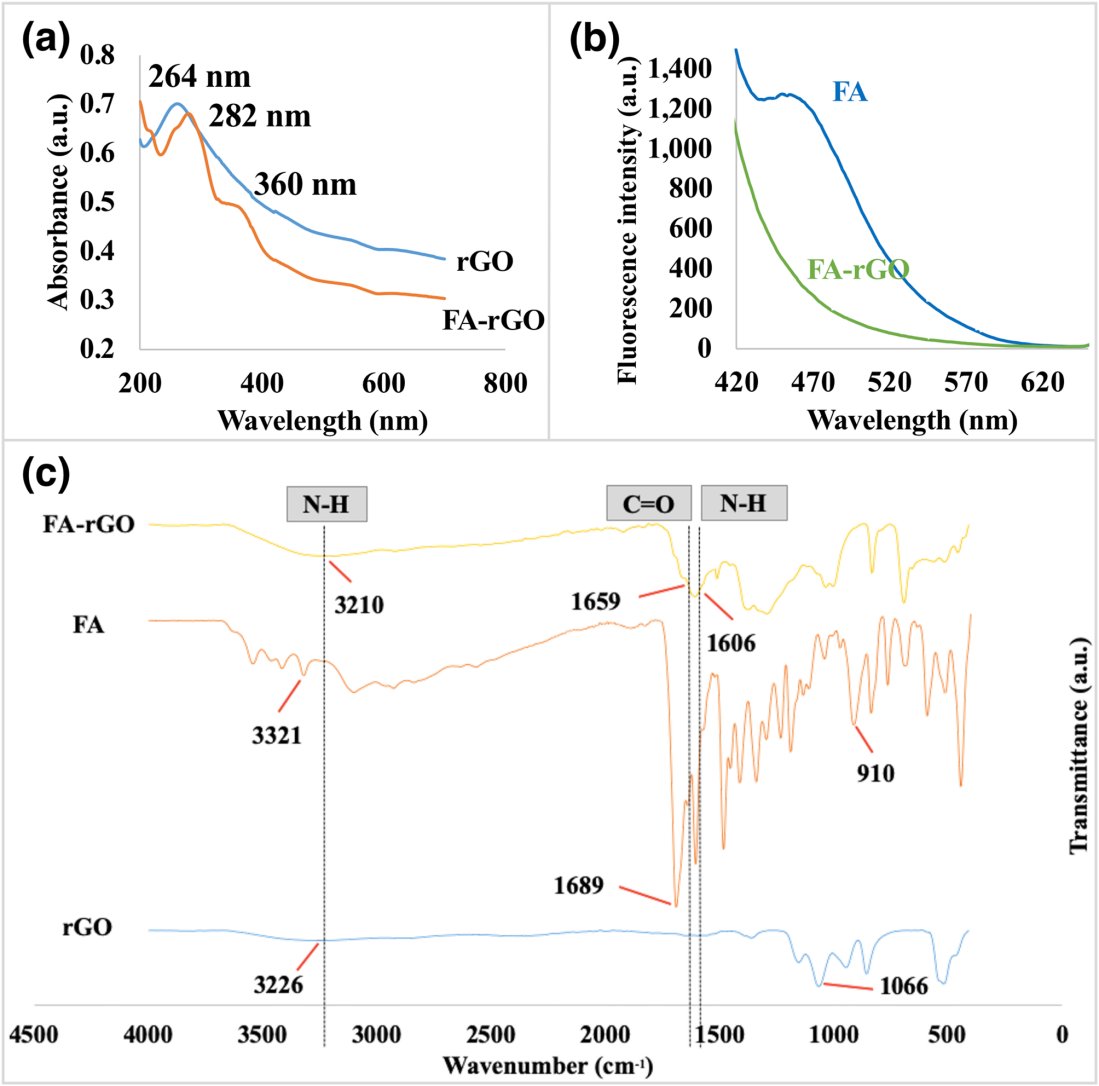
Characterization of FA-rGO. (**a**) UV–Vis spectra of rGO and FA-rGO in aqueous solution. (**b**) Fluorescence intensity of FA and FA-rGO at λ_ex_ = 365 nm. (**c**) FTIR spectra of FA, rGO and FA-rGO measured in lyophilized form.

In the UV–Vis spectra (Fig. 3a), the π–π* transition of pterin ring at 282 nm and the saddle point at 360 nm of FA were observed in FA-rGO, suggesting the conjugation of FA to rGO^56^. The FA-rGO exhibited characteristic absorption peaks of both FA and rGO. It can be observed that there is no fluorescence peak due to FA in the FA-rGO complex between 420 and 630 nm (Fig. 3b). In the FTIR spectrum of FA-rGO, the original peaks of FA at 3321 and 910 cm^−1^ belonging to O–H (stretching) disappeared. The peak at 1066 cm^−1^ corresponds to the carbonyl group (C–O) of rGO. The new peaks at 3210 (N–H stretching), 1659 (C=O stretching) and 1606 cm^−1^ (N–H bending) in the spectrum indicate the presence of CONH (amide) groups in the FA-rGO (Fig. 3c).

### Loading of BSA/AuNCs onto FA-rGO

**Figure 4:**
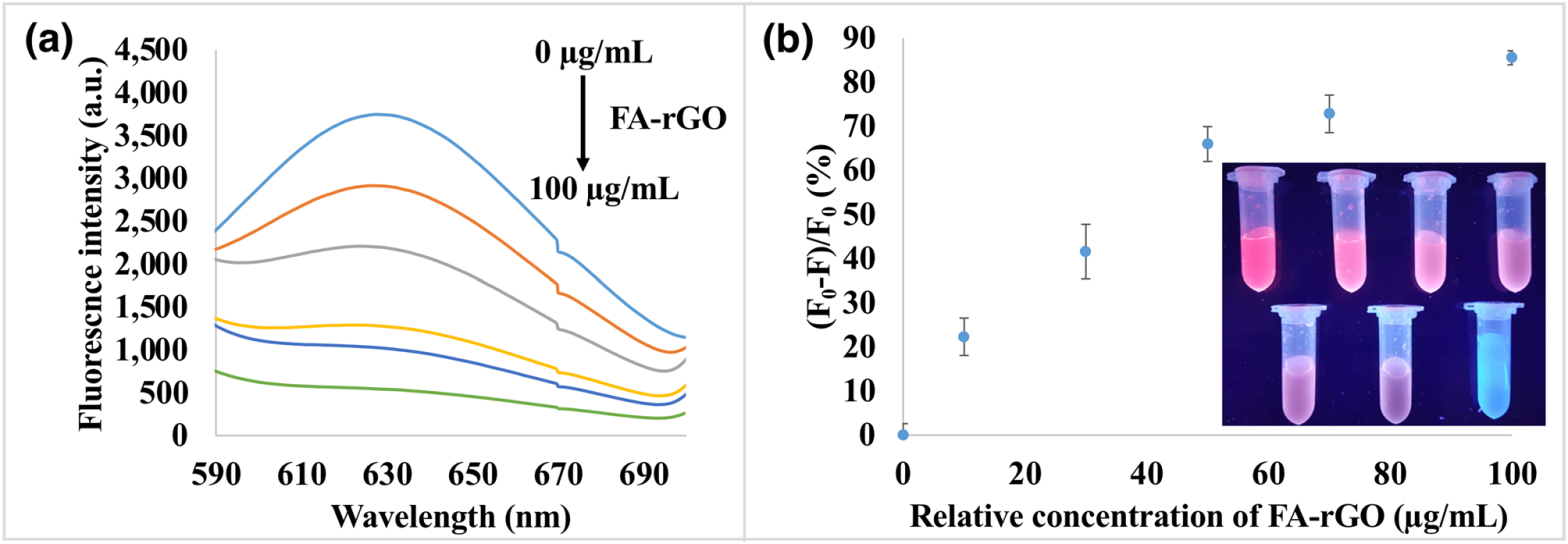
Effect of the addition of FA-rGO onto BSA/AuNCs. (**a**) Fluorescence intensity of 3 mg mL^−1^ of BSA/AuNCs at λ_ex_ = 365 nm, by varying the concentrations of FA-rGO. The relative concentrations of FA-rGO were 0, 10, 30, 50, 70 and 100 µg mL^−1^. (**b**) Fluorescence quenching values of 3 mg mL^−1^ of BSA/AuNCs by varying the concentrations of FA-rGO. Inset: optical photographs of BSA/AuNCs upon increasing the concentration of FA-rGO under UV light with λ_ex_ at 365 nm (from top left to down right). The last tube is the sample with FA-rGO only. Error bars indicate standard deviation of three independent measurements.

Conversion of ester, hydroxyl and epoxide groups in the rGO to carboxylic acid groups under strongly basic conditions may improve the aqueous stability of the reduced graphene sheets, and facilitate chemical binding of biomolecules via covalent bonding^42^. FA-rGO acts as a biocompatible biosensor for the detection of folate receptor-positive cancer cells. It is proposed that the binding of BSA/AuNCs onto FA-rGO was non-covalent, driven by hydrophobic interactions and π-π stacking between BSA/AuNCs and aromatic regions of the rGO sheets^67^. Interaction of BSA/AuNCs with either the metallic core, the stabiliser or the linkage between these two, might interfere with the fluorescence properties^68^. The charge transfer from BSA/AuNCs to FA-rGO weakens the Au–S bond between cysteine residues and the Au core, which in turn reduces charge transfer from BSA ligands to AuNCs, leading to the fluorescence quenching of AuNCs. As displayed in Fig. 4, the higher the concentrations of FA-rGO, the higher the fluorescence quenching of BSA/AuNCs. A relative concentration of 50 µg mL^−1^ of FA-rGO was chosen since the fluorescence of BSA/AuNCs was quenched by about 61%.

### Effect of addition of GSH on the fluorescence intensity of FA-rGO-BSA/AuNCs

Generally, GSH is found in the cytosol of cells where its concentration is in the range of 1–10 mM^69^. Despite with picomolar-level detection capacity, most of the current nanomaterial-based GSH biosensors (Table 1) present several drawbacks for wider biological applications, such as its potential toxicity and immunogenicity, reproducibility on the synthesis of nanomaterials, etc^70^. For example, quaraines dye, an organic fluorophore with advantages of lower photodamage, deeper tissue penetration and minimal fluorescence background, has been used for bioimaging and selective detection of GSH^71^. However, the dye is chemically fragile and prone to form non-fluorescent aggregates in biological media. Therefore, it is desirable to fabricate a GSH biosensor which is biocompatible and can detect at least milli-molar concentrations of GSH^72^.

**Table 1:** Examples of GSH biosensors with their sensitivity and linear range values as reported in the literature.

Schematic biosensor assembly	Signal	Linear range	Limit of detection	References
2D MnO_2_ nanosheets- BSA/AuNCs	Fluorescence	0–2 mM	20 µM	^5^
MnO_2_-induced synthesis of polydopamine NPs	Fluorescence	0–800 µM	1.5 µM	^18^
Bis-squaraine dye SQSS	Fluorescence	0–10 µM	0.15 µM	^71^
Carbon NPs@MnO_2_–AgNP nanocomposite	Fluorescence	0.8–80 µM	0.55 µM	^73^
Mesoporous silica nanoquenchers capped with anti-GSH antibody	Fluorescence	0–10 mM	52 pM	^72^
Carbon dot–MnO_2_ nanosheet	Fluorescence	0.2–600 µM	22 nM	^74^
AuNCs–Hg(II) system	Fluorescence	0.04–16.0 µM	7.0 nM	^75^
MoS_2_ quantum dot donor and Rhodamine 6G dye acceptor	Fluorescence	5–50 nM	2.7 nM	^76^
Self-quenched BSA/AuNCs	Fluorescence	0.1–1.5 mM	0.004 mM	^77^
BSA/AuNCs-MnO_2_ nanocomposite	Fluorescence	2–200 µM	2.2 µM	^78^
Arginine–glycine-aspartate (RGD)-modified BSA/AuNCs	Fluorescence	1–10 mM	Nil	^79^
Lucigenin-MnO_2_ nanosheets	Fluorescence	1–150 µM	180 nM	^24^
Poly (allylamine) hydrochloride-confined BSA/AuNCs	Fluorescence	0.3–20 mM	3 mM	^80^
Mixed-valence-state cobalt nanomaterials	Colorimetric	0.5–40 µM	0.03 µM	^21^
FA-rGO-BSA/AuNCs	Fluorescence	0–1.75 µM	0.1 µM	Present work

**Figure 5:**
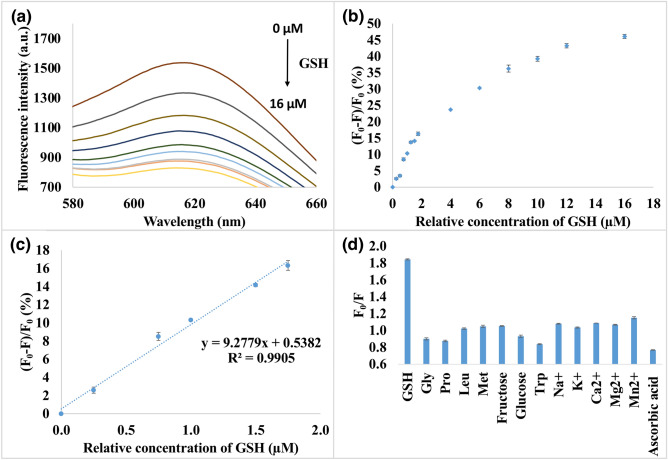
Sensitive and selective detection of GSH using FA-rGO-BSA/AuNCs. (**a**) Fluorescence intensity of FA-rGO- BSA/AuNCs by varying the concentrations of GSH. The relative concentrations of GSH were 0, 2, 4, 6, 8, 10, 12, 14 and 16 µg mL^−1^ at λ_ex_ = 365 nm. (**b**) Relationship between the fluorescence quenching values (F_0_ − F)/F_0_ and the target concentrations. (**c**) Linear response of the fluorescence quenching values (F_0_ − F)/F_0_ to the concentration of GSH. Error bars are the standard deviation of three repetitive experiments. (**d**) Selective detection of 0.5 mM GSH in the presence of potentially interfering components. The concentrations of glycine, proline, leucine, methionine, fructose, glucose, tryptophan, NaCl, KCl, CaCl_2_, MgSO_4_ and MnCl_2_·4H_2_O were 5 mM. The concentration of ascorbic acid was 50 mM. F_0_ and F are the fluorescence intensities of the sensing system in the absence and presence of GSH (or other potentially interfering chemicals), respectively. Error bars indicate the standard deviation of three independent measurements.

As depicted in Fig. 5, the addition of GSH into the mixture of FA-rGO and BSA/AuNCs gradually quenched its fluorescence. The fluorescence quenching values, (F_0_ − F)/F_0_, showed a linear dynamic range for the concentrations of GSH from 0.25 to 16 µM, which is sensitive to detect the milli-molar concentrations of endogenous GSH in most mammalian cells. As shown in the inset of Fig. 5c, a linear range from 0 to 1.75 µM as well as a LOD of 0.1 µM and a signal-to-noise ratio (SNR) of 7.68 towards GSH under the physiological pH conditions (pH 7.4) were obtained.

GSH has a sulfhydryl group and a glutamyl linkage in its structure^6^, making it a powerful reducing agent and a strong nucleophile that can react with cellular toxicants^81^. GSH plays the role of an antioxidant by scavenging electrophilic and oxidant species^1^. The possible mechanism contributing to the fluorescence quenching of FA-rGO-BSA/AuNCs could be due to the strong interaction (mainly by hydrogen bonding and van der Waals forces) between GSH and BSA on BSA/AuNCs. As a water-soluble biomolecule, BSA provides steric protection and shielding effect to AuNCs when acting as a fluorescent probe^37,82^. However, upon addition of GSH, driven by favourable enthalpy and unfavourable entrophy^6^, GSH binds within the sub-domain IIA pocket in domain II of BSA (as shown in Supplementary Fig. S3)^6^. This changes the conformation of BSA on BSA/AuNCs and forms a GSH-BSA complex^6^. The formation of GSH-BSA complex further destabilises the structure of FA-rGO-BSA/AuNCs and subsequently quenches the fluorescence intensity of BSA/AuNCs. Sensing strategies based on direct analyte-induced BSA/AuNCs fluorescence change can be simple but comes with the disadvantage of high sample matrix interference, especially in the detection of real samples^83^. This is because the analyte (in this case GSH) tends to interact with the Au core and ligands of BSA/AuNCs. The interaction may affect the valence state of Au core and form complexes, cluster aggregations or electron flow changes, which will eventually interfere with the fluorescence of BSA/AuNCs^84–86^. Therefore, special functionalization or modification of BSA/AuNCs are often needed for enhanced biosensing performances. Since covalent binding of FA to rGO can produce stable and biocompatible materials with potential as a nanocarrier in the drug delivery system^87^, this is the first study that explores using FA-rGO as a carrier for BSA/AuNCs in the detection of GSH.

To the best of our knowledge, there are no reports on the addition of GSH directly onto BSA/AuNCs. BSA/AuNCs are normally coupled with MnO_2_ nanosheets^78^, peptide^79^, polymer or subjected to growth process^80^ before using as an activatable fluorescence probe for GSH sensing. A possible justification for the modification of BSA/AuNCs was to enhance aurophilic interactions of Au(I)-thiolate complexes on the surface of Au(0) core and rigidify the ligand shell. This allows AuNCs to undergo aggregation-induced emission mechanism with enhanced fluorescence intensity^80^ while retaining the intrinsic structure of BSA/AuNCs surface and biological functions of BSA^78,79^. In addition, the surface modification of BSA/AuNCs reduces unwanted intramolecular vibration and rotation^80^, enhances biocompatibility and stability, as well as diversifies the potential of BSA/AuNCs in biological applications^79^.

A self-quenched BSA/AuNCs for turn-on fluorescence imaging of intracellular GSH was reported in 2017^77^. The self-quenched BSA/AuNCs were prepared via disulfide bond-induced aggregation of AuNCs. AuNCs act as both energy donor and acceptor. However, compared with the self-quenched AuNCs, the present work is much simpler and straightforward, exhibits higher quantum yield, a lower limit of detection (up to 40 times) and better selectivity over other interfering species. This study also eliminates ultrasonication and multi covalent coupling procedures.

Another possible reason for fluorescence quenching of FA-rGO-BSA/AuNCs could be ascribed to the formation of GSH-Au^+^ complexes via the specific etching reaction of the thiol group of GSH with the core of Au^88^. A similar observation has been reported in AgNCs, in which the biological thiols penetrate the BSA protective layer and chemisorb onto the surface of AgNCs, resulting in the fluorescence quenching of AgNCs^89^.

In the present study, the GSH nanobiosensor was designed based on the fluorescence “turn-off” strategy, in which fluorescence quenching occurred when GSH was added to the FA-rGO-BSA/AuNCs. Literature suggested that “turn-on” fluorescence strategy may provide more sensitive results with lower background signal and limit of detection^90,91^. Therefore, this work serves as a preliminary study for the design of “turn-on” fluorescence strategy with improved performance on selectivity and sensitivity. In future work, the effect of the addition of folate receptor, a promising cancer biomarker, on the FA-rGO-BSA/AuNCs can be investigated, which will lay the foundation for concurrent diagnosis and therapy of cancer cells.

### Selectivity of the sensing system

Selectivity is an essential parameter for probes in practical applications. The selectivity of the sensing system towards GSH detection over other amino acids and common components of metal ions was evaluated. As shown in Fig. 5d, the potential interfering compounds (glycine, proline, leucine, methionine, fructose, glucose, tryptophan, NaCl, KCl, CaCl_2_, MgSO_4_, and MnCl_2_·4H_2_O) with a concentration of ten times higher than the amount of GSH (5 mM vs 0.5 mM of GSH) did not significantly affect the detection. Notably, the fluorescence intensity of FA-rGO-BSA/AuNCs can be recovered in the presence of ascorbic acid with a concentration of 100 times higher than the amount of GSH (50 mM vs 0.5 mM of GSH). However, the interference of ascorbic acid could be eliminated by pre-treatment with *N*-ethylmaleimide (NEM, a thiol blocking agent)^92,93^. Despite the limitation of this approach towards antioxidant such as ascorbic acid, considering the concentration of GSH in cancer cells which is around 1–10 mM, the proposed sensing strategy exhibited good sensitivity and selectivity towards GSH detection.

## Conclusions

In this study, we have reported a novel nanobiosensor composed of BSA/AuNCs, rGO and FA. It is a new, fast and facile fabrication method of BSA/AuNCs, with high QYs under mild, economical and eco-friendly synthesis conditions. FA-rGO serving as an effective quencher towards the fluorescence of BSA/AuNCs has been demonstrated successfully, with the quenching intensity of about 61%. This is due to the effective charge transfer from BSA/AuNCs to rGO-FA, which weakens the Au–S bond between cysteine residues and the Au core of the initially fluorescent BSA/AuNCs complex. Furthermore, a sensitive and selective fluorescent sensing system for GSH detection was demonstrated based on the strong interaction between GSH and BSA on BSA/AuNCs. Our proposed method does not require an antibody and is more stable in time, biocompatible and uses a simpler and straightforward system that can be further developed into a visual/colorimetric sensor. This study assists in understanding the mechanisms of nanomaterial-mediated fluorescence quenching. It also paves the way to fabricate a “turn-on” or “turn-off” fluorescent nanobiosensor for relevant biomarkers in cancer cells, presenting potential nanotheranostic applications in biological detection and clinical diagnosis.

## Supplementary Information

Supplementary Information.
