# Supplementary material for: Integrating gold nanoclusters, folic acid and reduced graphene oxide for nanosensing of glutathione based on “turn-off” fluorescence
Source: Sci Rep. 2021 Jan 27;11:2375. doi: 10.1038/s41598-021-81677-8 (PMC7841173; doi:10.1038/s41598-021-81677-8)
Supplement: Supplementary file 1 — Supplementary Information. [file 41598_2021_81677_MOESM1_ESM.docx]

| Integrating gold nanoclusters, folic acid and reduced graphene oxide for nanosensing of glutathione based on “turn-off” fluorescence |
| --- |
| Xin Yi Wong1, Daniel Quesada-González2, Sivakumar Manickam1,3,4, Siu Yee New5, Kasturi Muthoosamy3* & Arben Merkoçi6,7*  1Department of Chemical & Environmental Engineering, Faculty of Science and Engineering, University of Nottingham Malaysia, 43500 Semenyih, Selangor, Malaysia  2Paperdrop Diagnostics, Av. de Can Domènech s/n, Eureka Building, Campus UAB – 08193, Bellaterra, Barcelona, Spain  3Nanotechnology Research Group, Centre of Nanotechnology and Advanced Materials, University of Nottingham Malaysia, 43500 Semenyih, Selangor, Malaysia  4Petroleum and Chemical Engineering, Faculty of Engineering, Universiti Teknologi Brunei, Bandar Seri Begawan, BE1410, Brunei Darussalam  5School of Pharmacy, Faculty of Science and Engineering, University of Nottingham Malaysia, 43500 Semenyih, Selangor, Malaysia  6Nanobioelectronics and Biosensors Group, Catalan Institute of Nanoscience and Nanotechnology (ICN2), CSIC, and The Barcelona Institute of Science and Technology (BIST), Campus UAB, Bellaterra, 08193 Barcelona, Spain  7ICREA, Institució Catalana de Recerca i Estudis Avançats, Pg. Lluis Companys 23, 08193 Barcelona, Spain  *****E-mail: [Kasturi.Muthoosamy@nottingham.edu.my;](mailto:Kasturi.Muthoosamy@nottingham.edu.my) [arben.merkoci@icn2.cat](mailto:arben.merkoci@icn2.cat)  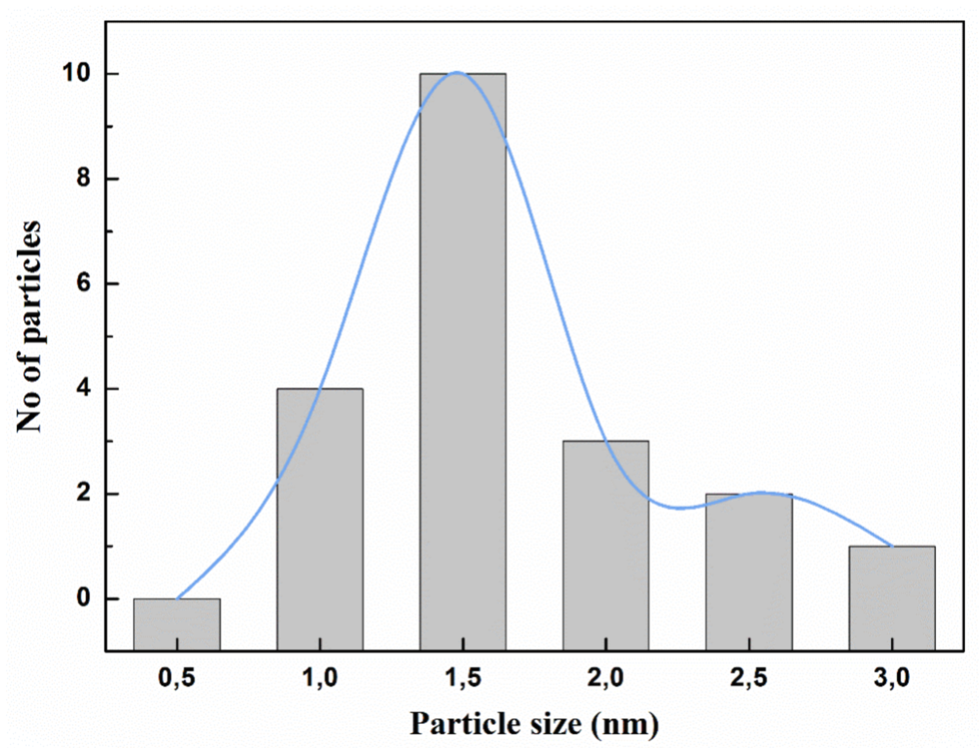 |

**Figure S1** Particle size distribution of BSA/AuNCs.


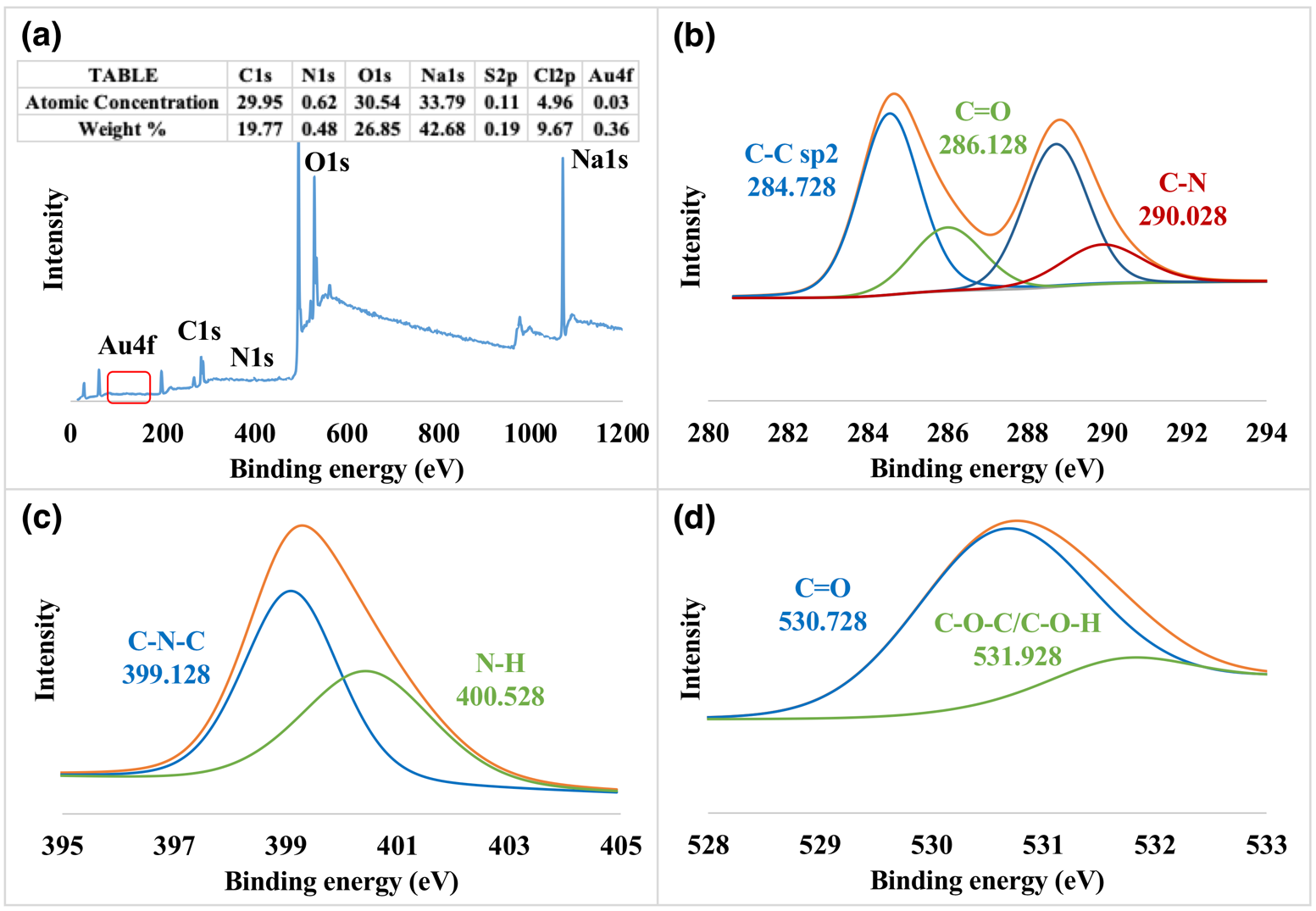


**Figure S2** Representative X-ray photoelectron spectroscopy (XPS) spectra of (a) full range, (b) C 1s, (c) N 1s, and (d) O 1s of BSA/AuNCs.


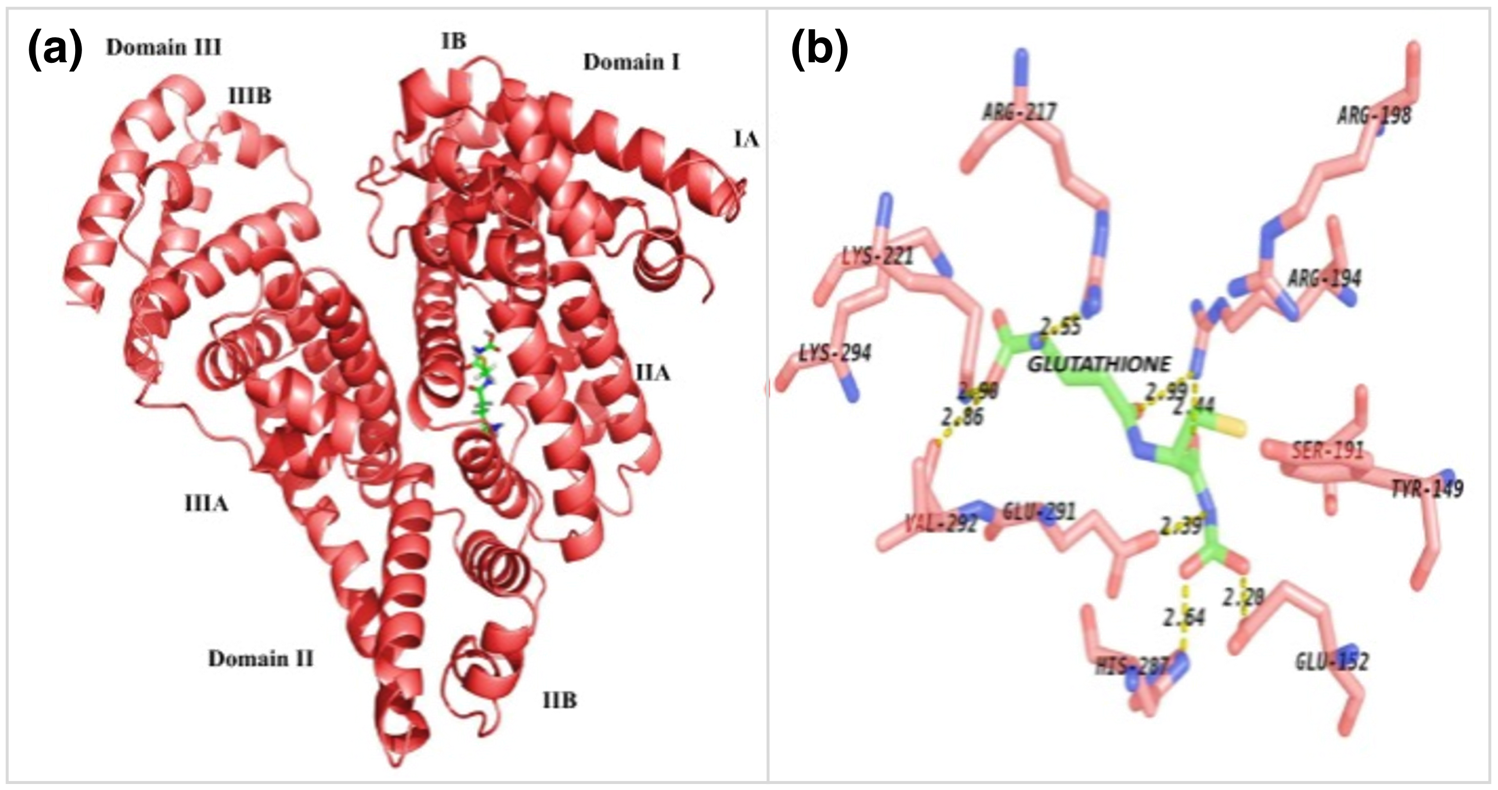


**Figure S3** Interaction of GSH with BSA. (a) Conformation of the binding mode between GSH and BSA. GSH binds within the site I of sub-domain IIA pocket in the domain II of BSA. (b) Hydrogen bonds between GSH and amino acid residues of BSA. Reprinted with permission from ref.1. Copyright Elsevier, 2016.

# References

1. Jahanban-Esfahlan, A. & Panahi-Azar, V. Interaction of glutathione with bovine serum albumin: spectroscopy and molecular docking. *Food Chemistry* **202**, 426–431 (2016).
